# Promoting Efficacy and Environmental Safety of Pesticide Synergists via Non-Ionic Gemini Surfactants with Short Fluorocarbon Chains

**DOI:** 10.3390/molecules27196753

**Published:** 2022-10-10

**Authors:** Ruiguo Wang, Xinxin Xu, Xiaodi Shi, Junjie Kou, Hongjian Song, Yuxiu Liu, Jingjing Zhang, Qingmin Wang

**Affiliations:** 1 State Key Laboratory of Elemento-Organic Chemistry, Research Institute of Elemento-Organic Chemistry, College of Chemistry, Frontiers Science Center for New Organic Matter, Nankai University, Tianjin 300071, China; 2 College of Basic Science, Tianjin Agricultural University, Tianjin 300392, China

**Keywords:** non-ionic gemini fluorinated surfactant, static/dynamic surface tension, diffusivity coefficient, wetting properties, contact angle, synergist

## Abstract

Improving the utilization rate of pesticides is key to achieve a reduction and synergism, and adding appropriate surfactant to pesticide preparation is an effective way to improve pesticide utilization. Fluorinated surfactants have excellent surface activity, thermal and chemical stability, but long-chain linear perfluoroalkyl derivatives are highly toxic, obvious persistence and high bioaccumulation in the environment. Therefore, new strategies for designing fluorinated surfactants which combine excellent surface activity and environmental safety would be useful. In this study, four non-ionic gemini surfactants with short fluorocarbon chains were synthesized. The surface activities of the resulting surfactants were assessed on the basis of equilibrium surface tension, dynamic surface tension, and contact angle. Compared with their monomeric counterparts, the gemini surfactants had markedly lower critical micelle concentrations and higher diffusivities, as well as better wetting abilities. We selected a single-chain surfactant and a gemini surfactant with good surface activities as synergists for the glyphosate water agent. Both surfactants clearly improved the efficacy of the herbicide, but the gemini surfactant had a significantly greater effect than the single-chain surfactant. An acute toxicity test indicated that the gemini surfactant showed slight toxicity to rats.

## 1. Introduction

With the increase of people’s requirements for healthy diet and living environment, it will be a great challenge for people to maintain the stable growth of crops in the next 30 years, as The United Nations Population Division estimates that there will be 10 billion people on Earth [1]. One essential solution to this impending food crisis is the extensive use of pesticides to increase agricultural productivity. However, the inefficiency of pesticides caused by spatter forces farmers to rely excessively on the overuse of pesticides. Wastage of pesticides cause substantial environmental pollution and human being health problems [2]. Therefore, it is of great important to enhance the deposition of agrochemical droplets on the surface of plant leaves. Surfactants can improve the deposition of agrochemical droplets on surfaces by reducing the surface tension and increasing the wettability of droplets on surfaces.

Unlike hydrocarbon surfactants, fluorinated surfactants have excellent surface activity and thermal and chemical stability [3], so have many potential applications [4,5,6,7,8,9,10,11,12]. Research on ionic gemini fluorinated surfactants has been published [13,14,15,16], but non-ionic versions have rarely been reported. The Tong group prepared two non-ionic gemini fluorocarbon surfactants containing ester bonds [17], these surfactants showed extremely low surface tensions, but because of their long fluorocarbon chains (nine CF_n_ groups), they were highly toxic and showed obvious persistence and high bioaccumulation in the environment [18,19,20]. Shortening the fluorocarbon chains has been shown to have potential utility for the development of slightly toxic fluorine-containing surfactants [21,22]. However, shortening the fluoroalkyl chains negatively affects the surface activity, which is based on conventional molecular structure design. Therefore, new strategies for designing short-chain fluorinated surfactants which combine excellent surface activity and environmental safety would be useful.

Gemini surfactants, also called dimeric surfactants, are composed of two hydrophobic chains and two hydrophilic groups that are covalently attached to a spacer such as a polymethylene chain; a heteroatomic chain containing nitrogen, oxygen, or sulfur atoms; or a rigid aromatic group [23,24,25]. Since the Bunton group synthesized a double quaternary ammonium bromide gemini surfactant in 1971, these surfactants have received increasing attention because of their unique properties [26]. Compared with conventional surfactants, gemini surfactants exhibit low CMCs, low Krafft temperatures, and better wettabilities, in addition to having unusual rheological properties [27].

Many syntheses of gemini hydrocarbon surfactants have been reported, but to our knowledge, there have been relatively few studies of gemini fluorinated surfactants. Moreover, research on the differences between flexible and rigid spacers and the differences between gemini fluorinated surfactants and their monomeric counterparts has not been reported either.

**Scheme 1:**
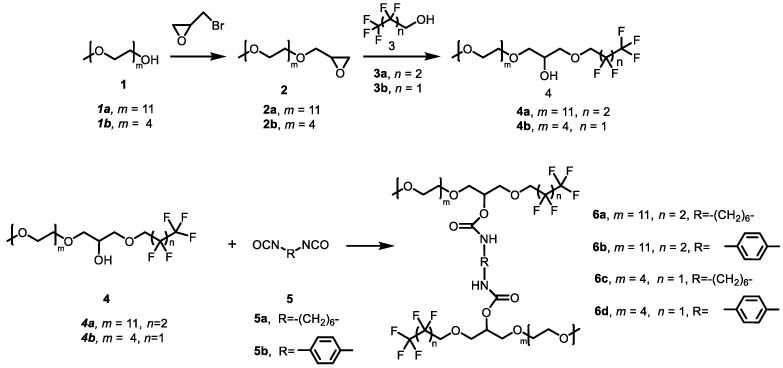
Synthesis of surfactants **4** and **6**.

In this work, we set out to design novel, environmentally safe non-ionic gemini surfactants with short fluorocarbon chains. First, environmentally friendly methoxypolyethylene glycols (mPEGs), epibromohydrin, and short-chain (≤3) perfluoroalkyl alcohols were used as raw materials for the synthesis of two single-chain surfactants. Second, four gemini surfactants with a diisocyanate spacer linking the hydroxyl groups of the single-chain surfactants were synthesized. Finally, the static and dynamic surface tension and the wetting properties of aqueous solutions of the surfactants were measured. Using the resulting data, we compared the effects of the flexible and rigid spacers and the differences between the gemini surfactants and their monomeric counterparts. Additionally, two surfactants with high surface activities (**4a** and **6a**, Scheme 1) were selected to test their efficacy as pesticide synergists. Finally, and most importantly, we assessed the acute toxicity of gemini surfactant 6a to rats to assess its environmental safety.

## 2. Results and Discussion

### 2.1. Synthesis of Surfactants and Determination of Their Structures

Single-chain fluorinated surfactants **4a** and **4b** were obtained by reactions of epibromohydrin with mPEGs **1** were followed by ring opening of the resulting mono-epoxide-functionalized mPEGs **2** with perfluoroalkyl alcohols **3** (Scheme 1). Gemini fluorinated surfactants **6a**–**6d** were obtained by reactions of single-chain fluorinated surfactants **4a** and **4b** with diisocyanates **5** (Scheme 1).

**Figure 1:**
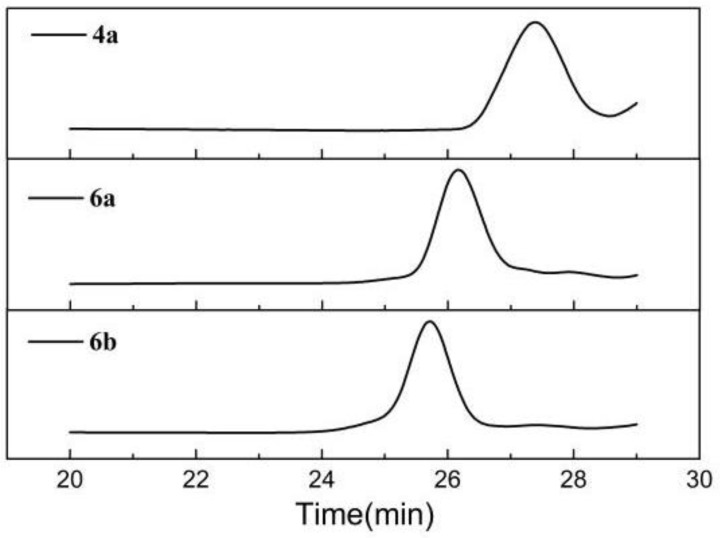
GPC spectra of **4a**, **6a**, and **6b**.

**Figure 2:**
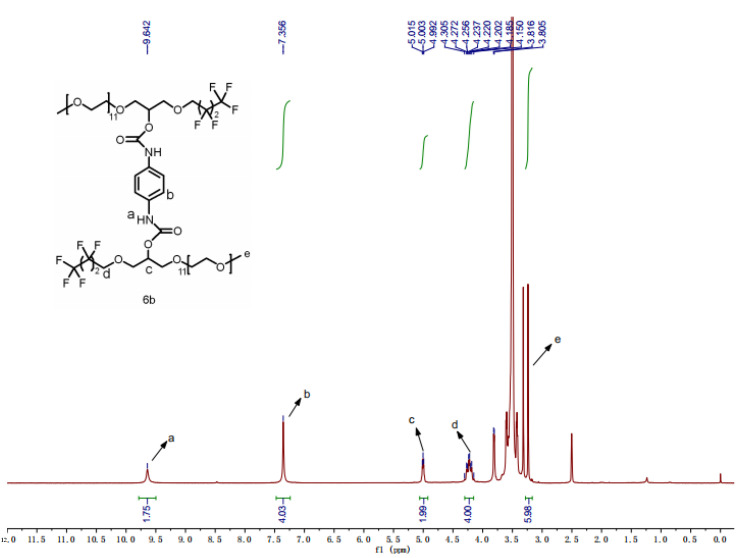
^1^H NMR spectrum of **6b**.

Because the reactivities of mPEGs depend on their molecular weights, we screened various 2/3 feed ratios and reaction temperatures and monitored the results by means of GPC and ^1^H NMR spectroscopy. The optimal conditions for the synthesis of each surfactant are listed in Table 1. In the GPC spectrum of **6b** (*m* = 11, *n* = 2; Figure 1) obtained under the optimized conditions, the retention time of the product peak was between 25 and 27.5 min, and the peak for **4a** (which had a longer retention time) was almost completely gone, indicating that **4a** had been almost completely consumed. In addition, in the ^1^H NMR spectrum of **6b** (Figure 2), the integration values for protons a–e were 1.75, 4.03, 1.99, 4.00, and 5.98, respectively; these values were consistent with the expected 2:4:2:4:6 ratio for these protons. The above-described results confirm that the desired product was obtained. The purities of **4a**, **6a**, and **6b** were determined by ^1^H NMR and ^19^F NMR spectroscopy (see Supporting Information).

**Figure 3:**
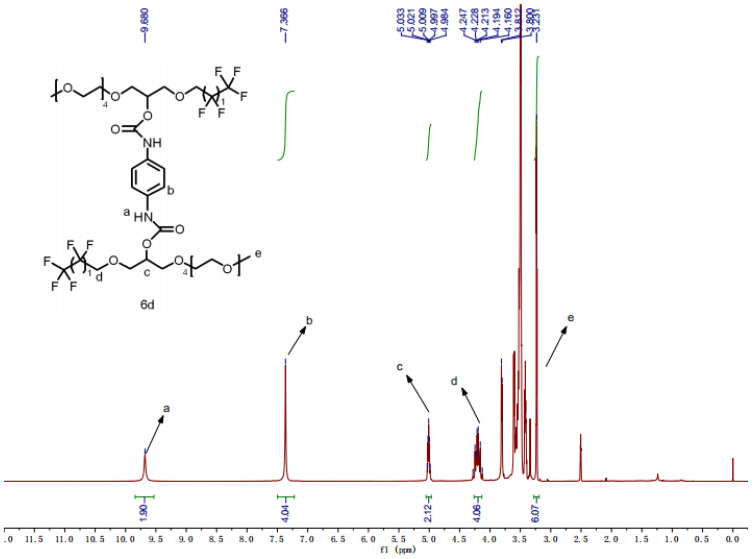
^1^H NMR spectrum of **6d**.

The structures of products with m = 4 were determined by comparing the ^1^H NMR spectrum of **4b** with the spectra of **6c** and **6d**. For example, the H(c) peak at δ 3.76 in the spectrum of **4b** (^1^H NMR spectrum, a peak that was assigned by DEPT-135 and HSQC spectroscopy [see Supporting Information]) was absent from the spectrum of **6d** (Figure 3), as was the H(a) peak at δ 4.96. Moreover, the chemical shifts and integration values for the protons in the spectrum of **6d** (Figure 3) were consistent with the proposed structure of this product, indicating that the reaction was successful. The purity of **6c** was determined by ^1^H NMR and ^19^F NMR spectroscopy (see Supporting Information).

### 2.2. Static Surface Tension

**Figure 4:**
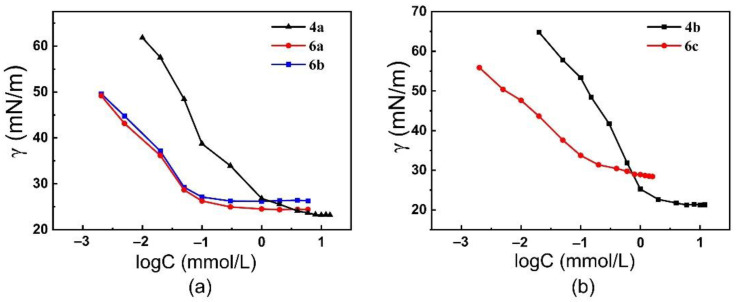
Concentration dependence of static surface tension of (**a**) **4a**, **6a**, and **6b** and (**b**) **4b** and **6c**.

We plotted surface tension (*γ*) versus the logarithm of bulk surfactant concentration (*C*, millimolar) to determine the CMC and the lowest surface tension (*γ*_min_) of each surfactant (representative plots are shown in Figure 4). In addition, from the surface tension curves, the adsorption efficiency (p*C*_20_) was obtained, which is the negative logarithm of the molar surfactant concentration necessary to lower the surface tension of water by 20 mN·m^−1^. We also calculated maximum surface excess concentrations (*Г*_max_) and minimum areas per surfactant molecule (*A*_min_) by using the Gibbs adsorption isotherm equations (Equations (1) and (2)) [28,29]:

(1) $$\Gamma_{\max}=\frac{-1}{2.303nRT}\left(\frac{\partial \gamma }{\partial \mathrm{lg}C}\right)$$

(2) $$A_{\min}=\frac{10^{18}}{N_{A}\Gamma_{\max}}$$

where *γ* is the surface tension of the surfactant solution (mN·m^−1^), *C* is the bulk concentration of the surfactant (mol/L), *n* is a constant (for non-ionic surfactants, *n* = 1), *R* is the gas constant, *T* is absolute temperature (K), and *N*_A_ is the Avogadro constant. The value of ∂*γ*/∂ lg *C* was determined from the slope of a linear fitting of the plot of surface tension versus lg *C* in the region below the CMC. The values determined from the plots and equations are shown in Table 2.

The surface tensions of solutions of **6a**, and **6b** decreased gradually with increasing surfactant concentration (Figure 4a). Comparing with **4a**, the surface tension of **6a** and **6b** decreased more quickly and reached its minimum value at a lower surfactant concentration. This indicated that the gemini surfactants had higher surface activities than their monomeric counterparts. The same trend was observed for **6c** (Figure 4b). Comparison of gemini surfactants **6a** and **6b**, which have different spacers, revealed that the surface tension of a solution of **6a** was lower than that of a solution of **6b** at the same concentration (Figure 4a). Although the differences were not particularly large, they demonstrate that the surface tension of gemini surfactants with a flexible spacer was lower than that of gemini surfactants with a rigid spacer, probably because of the ease of location of the flexible spacer at the water–air interface, which is consistent with previously reported results for hydrocarbon gemini surfactants [30].

The CMCs of the gemini surfactants were much lower than those of their monomeric counterparts (Table 2). For example, the CMC of single-chain surfactant **4a** was 3.31 mmol/L, whereas that of gemini surfactant **6a** was 0.110 mmol/L; that is, the CMC of **6a** was 1/30 that of **4a**. These results suggest that gemini surfactants readily formed aggregates. Aggregation may have been facilitated by strong hydrophobic synergism between the two hydrophobic chains of these surfactants [31]. The CMC of **6a** was similar to that of **6b**, indicating that the spacer was not the main determinant of CMC. Compared with single-chain surfactant **4a**, gemini surfactants **6a** and **6b** had slightly higher *γ*_min_ values. For example, the *γ*_min_ values of **4a** and **6a** were 23.31 and 24.43 mN/m. This difference may have resulted from gemini surfactants being more loosely arranged at the water–air interface than the single-chain surfactants. In addition, the *γ*_min_ of **6c** (*m* = 4) was larger than that of **4b** (28.89 versus 21.38 mN/m). Note that when the concentration of **6d** was higher than 0.1 mmol/L, the surfactant did not dissolve completely; and because the surface tension could not be balanced before 0.1 mmol/L, we could not calculate these important parameters for this surfactant. In addition, the maximum dissolved concentration of **6c** was only about 1.5 mmol/L. In other words, surfactants with an *m* value of 4 (**6c** and **6d**) showed poor solubility, which affected their surface tension.

The gemini surfactants had lower *Г*_max_ values and higher A_min_ values than their monomeric counterparts (Table 2). These results indicate that compared with the gemini surfactants, the single-chain surfactants tended to aggregate and arrange more closely at the water–air interface. The *Г*_max_ and *A*_min_ values of **6a**, which has a flexible spacer, were similar to those of **6b**, which has a rigid spacer. Compared with **6c**, **6a** had better solubility and a larger *Г*_max_, despite its higher molecular weight. This result once again shows that solubility strongly influenced surface activity. Generally, the adsorption efficiency of surfactant molecules at the water–air interface increases with increasing p*C*_20_ [32]. As shown in Table 2, the p*C*_20_ values of the gemini surfactants were larger than those of their monomeric counterparts; and the p*C*_20_ of **6a**, which has a flexible spacer, was higher than that of **6b**, which has a rigid spacer. These results show that **6a** had a greater preference for adsorption at the water–air interface than **6b** and could effectively reduce the surface tension of water.

### 2.3. Dynamic Surface Tension

**Figure 5:**
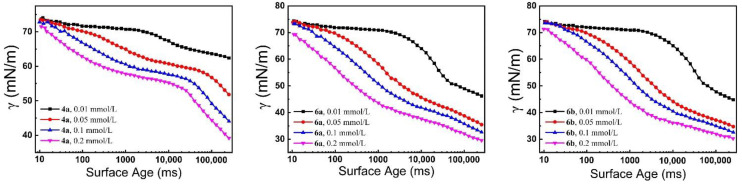
Surface-age-dependence of dynamic surface tension data for **4a**, **6a**, and **6b** at various concentrations.

**Figure 6:**
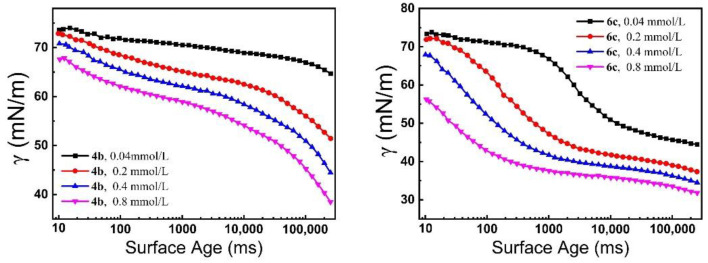
Surface-age-dependence of dynamic surface tension data for **4b** and **6c** at various concentrations.

To investigate the kinetics of adsorption of the fluorinated surfactants, the surface-age-dependence of the dynamic surface tension data for the three surfactants with *m* = 11 and *n* = 2 (**4a**, **6a**, and **6b**; Figure 5) and for the two surfactants with *m* = 4 and *n* = 1 (**4b**, **6c**; Figure 6) were evaluated. The plots indicated that as the surfactant concentration increased, both the rate and the magnitude of the reduction in dynamic surface tension increased.

The trends for gemini surfactants **6a** and **6b** were similar to each other (Figure 5); and surface tension dropped more quickly for these two surfactants than for the single-chain surfactant **4a**. For example, when the concentration of **6a** was 0.2 mmol/L, the surface tension was approximately 40 mN/m at 1000 ms, whereas the surface tension of **4a** at the same concentration was approximately 60 mN/m at 1000 ms. Moreover, both **6a** and **6b** (0.1 mmol/L) had a surface tension of 35 mN/m at 10,000 ms, whereas that of **4a** was approximately 55 mN/m at the same time.

As shown in Figure 6, at all concentrations, the surface tension of gemini surfactant **6c** quickly dropped to below the surface tension of water. For example, when the concentration of **6c** was 0.8 mmol/L, the surface tension was approximately 55 mN/m at 10 ms, whereas the surface tension of the single-chain surfactant **4b** was close to that of water at 10 ms. At a surface age of 100 ms, the surface tensions of **6c** and **4b** were approximately 40 and 60 mN/m, respectively. These results indicate that the structure of a surfactant is strongly affected its diffusion rate; gemini surfactants diffused rapidly at the water–air interface and were adsorbed more efficiently than their monomeric counterparts. The effects of surfactant structure on diffusion in aqueous solution will be discussed later.

Surfactant adsorption involves two sequential steps: (1) diffusion of surfactant molecules from the bulk aqueous phase to the subsurface and (2) absorption from the subsurface to the water–air interface [33]. The modified Ward–Tordai equation can be used to describe diffusion-controlled adsorption on a fresh surface. For the non-ionic surfactants in this study, we used the following simplified equation for the initial stage of adsorption [34,35,36]:

(3) $$\gamma {\left(t\right)}_{t\to 0}=\gamma_{0}-2RTC_{0}\sqrt{\frac{Dt}{\pi }}$$

where *γ*_0_ is the surface tension of ultrapure water (mN/m); *C*_0_ is the surfactant concentration (mol/L); and *D* is the diffusion coefficient of the surfactant.

The temporal dependence of surface tension for solutions of surfactants **4a**, **6a**, and **6b** at concentrations of 0.05, 0.1, and 0.2 mmol/L and for solutions of surfactants **4b** and **6c** at concentrations of 0.2 and 0.4 mmol/L (Figure 5 and Figure 6) were evaluated. These concentrations were chosen to ensure that the initial surface tension of the surfactant solution was >65 mN/m and that the adsorption layer was a dilute solution; if these conditions are not met, Equation (3) no longer applies [37]. In addition, the concentration must be high enough to minimize lateral diffusion of the surfactant from the thick surface to the thin surface of a generated air bubble, known as the Gibbs–Marangoni effect [38], causing the dominance of the vertical surfactant diffusion from the bulk liquid to the new surface of a generated air bubble.

**Figure 7:**
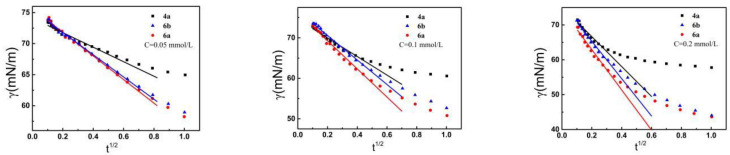
Linear behavior of *γ*(*t*)—*t*^1/2^ determined by the short time of surfactants **4a**, **6a**, and **6b**.

**Figure 8:**
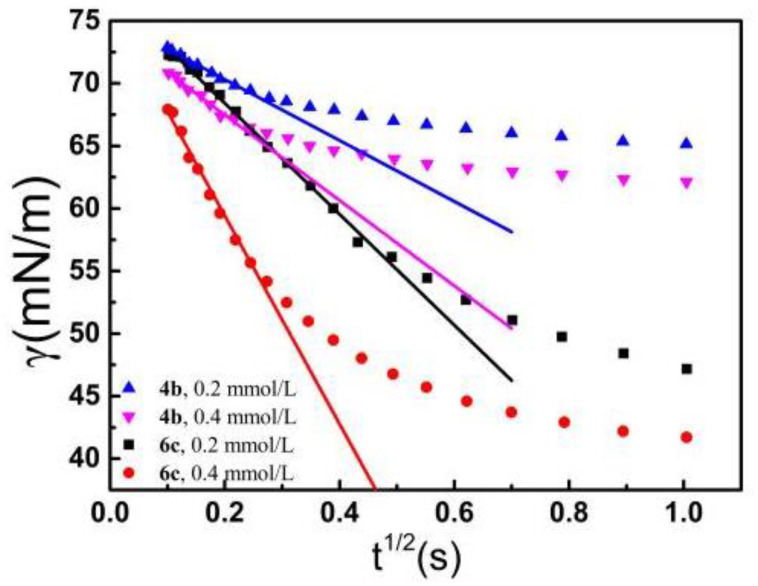
Linear behavior of *γ*(*t*)—*t*^1/2^ determined by the short time of **4b** and **6c**.

According to Equation (3), *γ*(*t*)(*t*→0) of a surfactant solution is linearly related to *t*^1/2^, so the *D* values of the surfactants can be determined from the slopes of the plots in Figure 7 and Figure 8; the values obtained in this way are listed in Table 3 and Table 4. During the initial stage of adsorption, the *D* values of gemini surfactants **6a**–**6c** were larger than the values of their monomeric counterparts (**4a** and **4b**), regardless of concentration. This result implies that **6a**–**6c** diffused faster and were adsorbed more effectively at the water–air interface than **4a** and **4b**. When the surfactant concentration was 0.2 mmol/L, the *D* values of both **6a** and **6b** were larger than the value of **6c**, indicating that **6a** and **6b**, which have relatively large mPEG molecular weights, diffused faster than **6c**. We also observed that the *D* value of **6a**, which has a flexible spacer, was similar to that of **6b**, which has rigid spacer. In addition, the *D* value for **6a** was slightly larger than that for **6b**, which is consistent with the surface tension measurements.

### 2.4. Wetting Properties

The wetting properties of surfactant solutions plays an important role in their applications [27,39], and contact angle is widely used as a metric for assessing surfactant wetting properties on low-energy solid surfaces [40]. Generally, the smaller the contact angle, the better the wetting ability.

**Figure 9:**
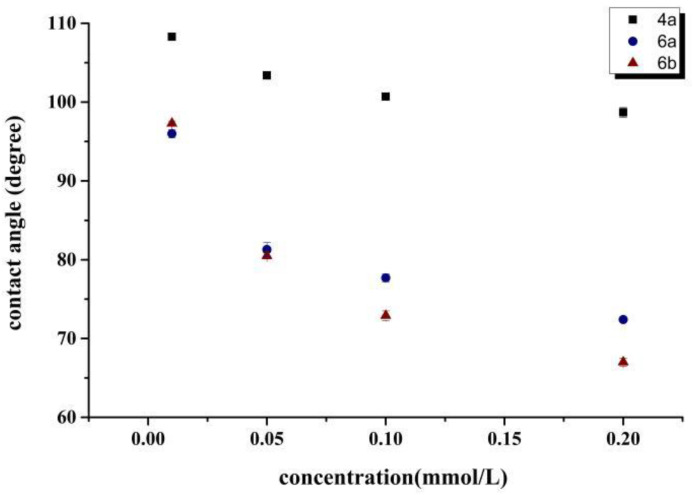
Contact angles of **4a**, **6a**, and **6b** at various concentrations.

**Figure 10:**
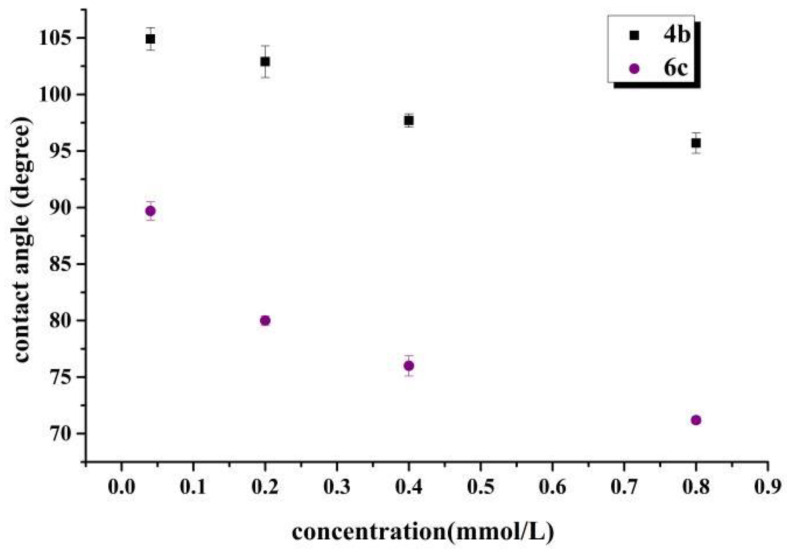
Contact angles of **4b** and **6c** at various concentrations.

The contact angles of aqueous solutions of gemini surfactants **6a**–**6c** and single-chain surfactants **4a** and **4b** on paraffin film were measured, which is a typical hydrophobic low-energy solid surface (Figure 9 and Figure 10). The measurements clearly revealed that the contact angles of **6a**–**6c** were lower than those of **4a** and **4b**, regardless of concentration. For example, at a concentration of 0.2 mmol/L, the contact angles of **6a** and **6b** were 72.4° and 67.0°, respectively, both of which were much smaller than the angle for **4a** (98.7°). These results indicate that the gemini surfactants had much better wetting properties than their monomeric counterparts. Comparison of the contact angles of **6a** and **6b**, which have different spacers, revealed they had similar wetting properties, which differs from that for previously reportedly hydrocarbon-based gemini surfactants [41]; our results indicate that the spacer was not the main determinant of wetting properties. At a concentration of 0.2 mmol/L, the contact angles of **6a** and **6b** were smaller than that of **6c** (80.0°), indicating that aqueous solutions of **6a** and **6b** had better wetting properties than solutions of **6c**.

### 2.5. Influence of Surfactants ***4a*** and ***6a*** on Glyphosate Water Agent

Because crop stems and leaves have waxy, hydrophobic surface layers and because many pesticides are not very hydrophilic, adding surfactants to pesticide formulations can improve the wettability, adhesion, spreading, and permeability of pesticide spray droplets, thereby improving pesticide efficacy [42,43,44,45]. To evaluate the efficacy of our surfactants for this purpose, we carried out some experiments with **4a** and **6a** as potential synergists for glyphosate water agent (roundup, active ingredient content: 30%), an effective, low-toxicity herbicide that is widely used to control the growth of weeds in agriculture.

**Figure 11:**
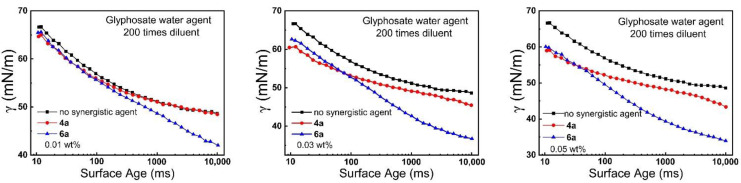
Dynamic surface tension data for aqueous solutions of glyphosate water agent containing surfactants **4a** or **6a**.

Static and dynamic surface tension values of aqueous solutions of glyphosate water agent, (diluted 200-fold) containing each of the surfactants at a concentration of 0.01, 0.03, or 0.05 wt% were determined. In the absence of surfactants, the static surface tension of aqueous glyphosate water agent, was 44.75 mN/m, but the surface tension values of herbicide solutions containing a surfactant were substantially lower (Table 5). Gemini surfactant **6a** had a greater effect on surface tension than the single-chain surfactant **4a** at all concentrations, but the difference between the two surfactants was most obvious at the lowest concentration. The gemini surfactant **6a** reduced surface tension faster than the single-chain surfactant **4a** at all concentrations (Figure 11). For example, at a surfactant concentration of 0.03 wt%, the surface tension of a solution containing **6a** was approximately 40 mN/m at 1000 ms, whereas the surface tension of a **4a**-containing solution was approximately 50 mN/m at 1000 ms. The lower the static surface tension, the easier the solution spreads on leaf surfaces; and easier spreading in turn increases the contact area between the solution and the leaves and improves the utilization of the pesticides [46,47,48].

**Figure 12:**
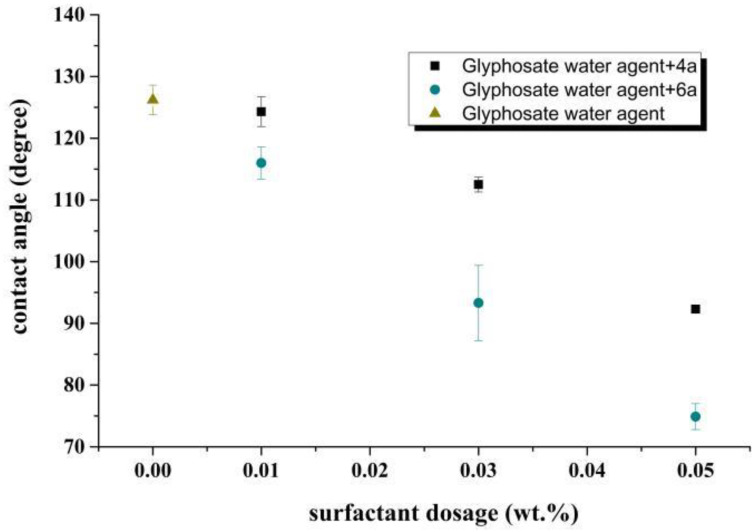
Contact angles of glyphosate water agent solutions containing surfactants **4a** or **6a**.

The contact angle of an aqueous solution of glyphosate water agent on corn leaves was 126.2° in the absence of surfactant. When surfactants were present, the contact angle was smaller (Figure 12), and gemini surfactant **6a** was more effective at decreasing the contact angle than the single-chain surfactant **4a**. For example, when the surfactant concentration was 0.03%, the contact angle of the solution containing **6a** was 93.3°, whereas the angle for the **4a-**containing solution was 112.5°. These results indicate that the addition of surfactants effectively reduced the surface tension of aqueous solutions of glyphosate water agent and can thus be expected to improve the diffusion and wettability of spray deposits on the surfaces of crop stems and leaves.

### 2.6. Evaluation of Acute Toxicity

Gemini surfactants are widely used in various fields, so evaluating their toxicity is important for assessing their environmental safety. In this study, the acute toxicity of **6a** to rats at dosages of 50 and 500 mg/kg were measured. We found that even at 500 mg/kg, **6a** caused no symptoms of poisoning in the rats and that there was no significant difference in the weights of treated animals and untreated controls. Upon dissection, none of the animals showed any abnormalities visible to the naked eye. These results indicate that **6a** has low toxicity and is thus likely to be safe to use. Our research results overcome the shortcomings of the high toxicity of traditional fluorosurfactants and provide a reference for subsequent research on low-toxicity fluorosurfactants [18,19,20].

## 3. Materials and Methods

### 3.1. Chemicals and Instruments

mPEG-500 was purchased from Macklin Co. (Shanghai, China), and mPEG-200 was purchased from 3A Co. (Shanghai, China). 1H,1H-perfluoro-1-propanol was purchased from J&K Co. (Beijing, China, purity:97%), and 1H,1H-perfluoro-1-butanol was purchased from Meryer Co. (Shanghai, China, purity:98%). 1-Bromo-2,3-epoxypropane (purity:97%) 1,4-phenylene diisocyanate (purity:98%) and hexamethylene diisocyanate (purity:99%) were purchased from J&K Co. (Beijing, China). Glyphosate-isopropylammonium 41% aqueous solution (AS) was purchased from Syngenta Nantong Crop Protection Co. Ultrapure water was purified by UPR-II-5T (ULUPURE, Chengdu, China). All reagents were used as received. All the reactions were performed under argon. ^1^H NMR, DEPT-135 (distortionless enhancement polarization transfer), HSQC (heteronuclear single quantum coherence), and ^19^F NMR spectra were recorded on a Bruker AV 400 spectrometer. Surfactant molar masses and molar mass distributions were determined by gel permeation chromatography (GPC) on a Waters 1525 instrument with THF as the mobile phase.

### 3.2. Surface Tension Measurements

Static surface tension values for aqueous solutions were measured by means of a platinum ring test at 25 ± 1 °C on an automatic processor tensiometer (JK99M). Reported values are averages of three measurements. Dynamic surface tension values were measured by means of the maximum bubble pressure method at 25 ± 1 °C on a Kruss BP100 tensiometer. Both instruments were calibrated with ultrapure water prior to sample analysis.

### 3.3. Contact Angle Measurements

The wetting abilities of aqueous solutions of the surfactants on a paraffin film were investigated by measuring contact angles at 25 °C using the sessile drop method with an OCA 25 drop shape analyzer (Dataphysics Co., Filderstadt, Germany). When measuring the contact angles of glyphosate water agent aqueous solutions containing surfactant **4a** or **6a** as synergists, we used corn leaves as the solid substrate. Each sample was repeated three times, and the average value was obtained.

### 3.4. Acute Toxicity to Rats

Acute toxicities to rats were determined by Xu He (Tianjin) Pharmaceutical Technology Co. The experimental rats were divided into two groups, which received the surfactant orally at doses of 50 and 500 mg/kg, respectively; the rats were fasted the day before surfactant administration. Depending on the effects of the surfactant on the rats, the compounds were classified as being extremely toxic (LD_50_ < 5 mg/kg), highly toxic (5 ≤ LD_50_ < 50 mg/kg), moderately toxic (50 ≤ LD_50_ < 500 mg/kg), or slightly toxic (LD_50_ ≥ 500 mg/kg).

## 4. Conclusions

Four non-ionic gemini surfactants with short fluorocarbon chains (≤3) were synthesized and characterized by ^1^H NMR, DEPT-135, HSQC, and ^19^F NMR spectroscopy, as well as GPC. Compared with single-chain surfactants, gemini surfactants have slightly higher γ_min_ values. However, measurements of CMCs, dynamic surface tension, and wetting ability revealed that the surface activities of the gemini surfactants were substantially better than those of their monomeric counterparts; the most obvious feature was the extremely low CMCs of the gemini surfactants. As a representative, addition of **6a** effectively reduced the surface tension of aqueous solutions of glyphosate water agent and improved the diffusion and wettability of spray deposits on the surface of crop leaves. The acute toxicity evaluation results indicated that **6a** had low toxicity and was thus likely to be safe to use. All of those indicated that non-ionic gemini surfactants with short fluorocarbon chains showed potential as a pesticide synergist. In light of our findings, our synthetic approach to these low-CMC gemini surfactants can be expected to find utility for the development of emulsifiers, paints, and cosmetics, as well as drugs and other biologically active compounds.

## Figures and Tables

**Table 1:** Reaction conditions for the synthesis of surfactants **4** and **6**.

Entry	2	3	2/3	T (°C) ^a^	4	4/5	T (°C) ^b^	Solvent ^c^	6
1	**2a**	**3a**	1:4	70	**4a**	2:1	110	toluene	**6a, 6b**
2	**2b**	**3b**	1:3	50	**4b**	2:1	90	dichloroethane	**6c, 6d**

^a^ Temperatures for the reactions of 2 and 3 in the presence of t-BuOK (1 equiv) for 24 h. ^b^ Temperatures for the reactions of 4 and 5; the reaction time was 12 h. ^c^ Solvent for the reactions of 4 and 5.

**Table 2:** Values of CMC, *γ*_min_, *Г*_max,_ *A*_min_, and p*C*_20_ for the surfactants.

Surfactant	*n*	*m*	CMC ^a^ (mmol/L)	*γ*_min_ (mN/m) ^b^	*Г*_max_ (mol/m^2^) ^c^	*A*_min_ (nm^2^) ^d^	p*C*_20_
**4a**	2	11	3.310	23.31	2.65 × 10^−3^	6.27 × 10^−4^	1.49
**6a**	2	11	0.110	24.43	2.42 × 10^−3^	6.86 × 10^−4^	2.81
**6b**	2	11	0.105	26.31	2.43 × 10^−3^	6.83 × 10^−4^	2.78
**4b**	1	4	2.450	21.38	3.68 × 10^−3^	4.51 × 10^−4^	0.96
**6c**	1	4	0.407	28.89	1.88 × 10^−3^	8.83 × 10^−4^	2.47
**6d**	1	4	—	34.79 ^e^	—	—	

^a^ Critical micelle concentration. ^b^ Lowest surface tension measured. ^c^ Maximum surface excess concentration. ^d^ Minimum area per surfactant molecule. ^e^ Surface tension of 6d at 0.1 mmol/L.

**Table 3:** Diffusion coefficients of surfactants **4a**, **6a**, and **6b**.

*C* (mmol/L)	Surfactant	*D* (m^2^/s)
0.05	**4a**	7.01 × 10^−9^
	**6a**	1.85 × 10^−8^
	**6b**	1.64 × 10^−8^
0.1	**4a**	6.74 × 10^−9^
	**6a**	1.55 × 10^−8^
	**6b**	1.17 × 10^−8^
0.2	**4a**	5.89 × 10^−9^
	**6a**	1.07 × 10^−8^
	**6b**	9.21 × 10^−9^

**Table 4:** Diffusion coefficients of **4b** and **6c**.

*C* (mmol/L)	Surfactant	*D* (m^2^/s)
0.2	**4b**	1.90 × 10^−9^
	**6c**	6.28 × 10^−9^
0.4	**4b**	9.32 × 10^−10^
	**6c**	5.54 × 10^−9^

**Table 5:** Static surface tension data for aqueous solutions of glyphosate water agent containing surfactants **4a** or **6a**.

Surfactant	0.01 wt%	0.03 wt%	0.05 wt%
**4a**	33.99 ± 0.30	29.61 ± 0.43	28.30 ± 0.30
**6a**	28.50 ± 0.15	26.70 ± 0.04	25.66 ± 0.40

## Data Availability

Not applicable.

## Supplementary Materials

The following supporting information can be downloaded at: https://www.mdpi.com/article/10.3390/molecules27196753/s1, Figure S1: ^1^H NMR of **4b**; Figure S2: HSQC spectrum of **4b**; Figure S3: Dept 135 spectrum of **4b**; Figure S4: ^1^H NMR of **4a**; Figure S5: ^19^F NMR of **4a**; Figure S6: ^1^H NMR of **4b**; Figure S7: ^19^F NMR of **4b**; Figure S8: ^1^H NMR of **6a**; Figure S9: ^19^F NMR of **6a**; Figure S10: ^1^H NMR of **6b**; Figure S11: ^19^F NMR of **6b**; Figure S12: ^1^H NMR of **6c**; Figure S13: ^19^F NMR of **6c**; Figure S14: ^1^H NMR of **6d**; Figure S15: ^19^F NMR of **6d**. Ref. [49] is cited in supplementary materials.
