# Supplementary material for: Promoting Efficacy and Environmental Safety of Pesticide Synergists via Non-Ionic Gemini Surfactants with Short Fluorocarbon Chains
Source: Molecules. 2022 Oct 10;27(19):6753. doi: 10.3390/molecules27196753 (PMC9572613; doi:10.3390/molecules27196753)
Supplement: Supplementary file 1 [file molecules-27-06753-s001.zip › molecules-1931237-supplementary.pdf]

molecules

Support information

Promoting Efficacy and Environmental Safety of Pesticide Synergists via Non-ionic Gemini Surfactants with Short Fluorocarbon Chains

Ruiguo Wang [2], Xinxin Xu [2], Xiaodi Shi [2], Junjie Kou [2], Hongjian Song [2,*], Yuxiu Liu [2], Jingjing Zhang [1,2,*] and Qingmin Wang [2,*]

[1] College of Basic Science, Tianjin Agricultural University, Tianjin 300392, China
[2] State Key Laboratory of Elemento-Organic Chemistry, Research Institute of Elemento-Organic Chemistry, College of Chemistry, Frontiers Science Center for New Organic Matter, Nankai University, Tianjin 300071, China
* Correspondence: songhongjian@nankai.edu.cn (H.S.); jingjingzhang216@outlook.com (J.Z.); wangqm@nankai.edu.cn (Q.W.); Tel.: +86-(0)22-23503952 (H.S.); Fax: +86-(0)22-23503952

Contents

1. General procedure and characterization for the preparation of **3a** and **3b** ……… S3

2. General procedure and characterization for the preparation of **4a** and **4b** ……… S3-S4

3. General procedure and characterization for the preparation of **6a-6d** ……… S4-S5

4. Determination of H(c) chemical shift of **4b** ……… S6-S7

5. ^{1}H NMR and ^{19}F NMR spectra of fluorinated Gemini surfactants (**4a**, **4b**, **6a-6d**) ……… S8-S13

6. References ……… S14

1. Synthesis of Mono-Epoxide-Functionalized mPEG (3a and 3b) (Wang et al., 2021)

Synthesis of 3a. In a 100 mL three-necked flask were added mPEG-500 (m = 11, 4.00 g, 8 mmol), sodium hydride (0.48 g, 60% content, 12 mmol), tetrahydrofuran (50 mL), and stirred at room temperature for 10 min, then epibromohydrin (1.63 g, 12 mmol) was added to the reaction solution, and the reaction mixture continued stirring for 24 h at room temperature under argon atmosphere. The reaction solution was filtered, tetrahydrofuran was evaporated in vacuo, and the residue was purified by column chromatography on silica gel with dichloromethane, methanol and ammonia hydroxide (20:1:0.04, v/v) as eluent to give **3a** as a light-yellow liquid, 3.20 g, 72% yield. ^{1}H NMR (400 MHz, $CDCl_3$) *δ* 3.80 – 3.40 (br, CH_2CH_2O), 3.38 (s, 3H), 3.20 – 3.13 (m, 1H), 2.82 – 2.75 (m, 1H), 2.65 – 2.58 (m, 1H).

Data for 3b. A similar process was conducted as **3a**. Column chromatography (silica gel, dichloromethane/methanol/ammonia hydroxide: 30/1/0.06, v/v), and this compound was obtained as a light-yellow liquid in 76% yield. ^{1}H NMR (400 MHz, $CDCl_3$) δ 3.83 – 3.41 (br, CH_2CH_2O), 3.38 (s, 3H), 3.20 – 3.12 (m, 1H), 2.83 – 2.76 (m, *J* = 4.5 Hz, 1H), 2.64 – 2.58 (m, 1H).

2. Synthesis of single chain fluorinated surfactants (4a and 4b)

Synthesis of single chain surfactant 4a. To a 100 mL three-necked flask were added 1H,1H-perfluoro-1-butanol (2.88 g, 14.4 mmol), potassium *tert*-butoxide (0.40 g, 3.60 mmol), and dioxane (20 mL). After stirring for 30 min, mono-epoxide-functionalized mPEG-500 (**3a**, 2.00 g, 3.60 mmol) dissolved in dioxane (20 mL) was added into the reaction solution. The reaction was heated at 70°C for 24 h under argon atmosphere. The reaction solution was filtered, water (0.1 mL) was added to quench the potassium alcohol, dried over anhydrous Na_2SO_4, and concentrated in vacuo. Then the residue was purified by column chromatography on silica gel with dichloromethane, methanol, and ammonia hydroxide as eluent (20:1:0.04, v/v) to offer **4a** as a light-yellow liquid 1.80 g in 66% yield. ^{1}H NMR (400 MHz, DMSO-d_6) δ 4.97 (d, *J* = 5.2 Hz, 1H), 4.19 (t, *J* = 14.4 Hz, 2H), 3.75 – 3.35 (br, CH_2O, $CHCH_2O$), 3.24 (s, 3H). ^{19}F NMR (376 MHz, DMSO-d_6) δ -80.52 (t, *J* = 9.8 Hz, 3F, CF_3), -119.81 – -120.10 (m, 2F, $CF_{2\alpha}$), -127.24 (s, 2F, $CF_{2\beta}$).

Data for single chain surfactant 4b. A similar process was conducted as **4a**, and this compound was obtained as a light-yellow liquid in 78% yield. ^{1}H NMR (400 MHz, DMSO-d_6) δ 4.97 (d, *J* = 5.2 Hz, 1H), 4.16 (t, *J* = 14.0 Hz, 2H), 3.62 – 3.31 (br, CH_2O and $CHCH_2O$), 3.24 (s, 3H). ^{19}F NMR (376 MHz, DMSO-d_6) δ -82.82 (s, 3F, CF_3), -122.66 – -122.95 (m, 2F, $CF_{2\alpha}$).

3. Synthesis of fluorinated Gemini surfactants (6a-6d)

Synthesis of Gemini surfactant 6a: To a 100 mL three-necked flask were added single chain surfactant **4a** (2.00 g, 2.65 mmol), hexamethylene diisocyanate (0.22 g, 1.32 mmol), toluene (25 mL). The reaction was carried out under reflux conditions for 12 h. The reaction requires strict anhydrous and oxygen-free. The reaction solution was filtered and concentrated in vacuo. Then the residue was purified by column chromatography on silica gel with dichloromethane, methanol, and ammonia hydroxide as eluent (15:1:0.03, v/v) to offer **6a** as a light-yellow liquid 2.05 g in 92% yield. ^{1}H NMR (400 MHz, DMSO-d_6) δ 7.24 (s, 2H), 4.81 – 4.89 (m, 2H), 4.28 – 4.08 (m, 4H), 3.80 – 3.32 (br, CH_2O, $CHCH_2O$), 3.24 (s, 6H), 2.94 (s, 4H), 1.36 (s, 4H), 1.22 (s, 4H). ^{19}F NMR (376 MHz, DMSO-d_6) δ -80.61 (t, *J* = 9.4 Hz, 3F, CF_3), -119.85 – -120.15 (m, 2F, $CF_{2\alpha}$), -127.30 (s, 2F, $CF_{2\beta}$).

Data for Gemini surfactant 6b. A similar process was conducted as **6a**, and this compound was obtained as a light-yellow liquid in 91% yield. ^{1}H NMR (400 MHz, DMSO-d_6) δ 9.64 (s, 2H), 7.36 (s, 4H), 5.04 – 4.96 (m, 2H), 4.30 – 4.15 (m, 4H), 3.75 – 3.31 (br, CH_2O, $CHCH_2O$), 3.24 (s, 6H). ^{19}F NMR (376 MHz, DMSO-d_6) δ -80.59 (t, *J* = 9.8 Hz, 3F, CF_3), -119.86 – -120.11 (m, 2F, $CF_{2\alpha}$), -127.28 (s, 2F, $CF_{2\beta}$).

Synthesis of Gemini surfactant 6c: To a 100 mL three-necked flask were added single chain surfactant **4b** (2.00 g, 4.92 mmol), hexamethylene diisocyanate (0.41 g, 2.46 mmol), dichloroethane (25 mL). The reaction was carried out under reflux conditions for 12 h. The reaction requires strict anhydrous and oxygen-free. The reaction solution was filtered and concentrated in vacuo. Then the residue was purified by column chromatography on silica gel with dichloromethane, methanol, and ammonia hydroxide as eluent (30:1:0.06, v/v) to offer **6c** as a light-yellow liquid 2.26 g in 94% yield. ^{1}H NMR (400 MHz, DMSO-*d$_6$*) δ 7.25 (t, *J* = 5.6 Hz, 2H), 4.92 – 4.81 (m, 2H), 4.24 – 4.05 (m, 4H), 3.76 – 3.31 (br, CH_2O, $CHCH_2O$), 3.25 (s, 6H), 3.05 – 2.86 (m, 4H), 1.38 (s, 4H), 1.23 (s, 4H). ^{19}F NMR (376 MHz, DMSO-*d$_6$*) δ -82.86 (s, 3F, CF_3), -122.84 (s, 2F, $CF_{2\alpha}$).

Data for Gemini surfactant 6d. A similar process was conducted as **6c**, and this compound was obtained as a light-yellow liquid in 90% yield. ^{1}H NMR (400 MHz, DMSO-*d$_6$*) δ 9.68 (s, 2H), 7.37 (s, 4H), 5.05 – 4.96 (m, 2H), 4.30 – 4.11 (m, 4H), 3.88 – 3.29 (br, CH_2O, $CHCH_2O$), 3.23 (s, 6H). ^{19}F NMR (376 MHz, DMSO-*d$_6$*) δ -82.78 (s, 3F, CF_3), -122.23 – -123.01 (m, 2F, $CF_{2\alpha}$).

4. Determination of H(c) chemical shift of 4b.

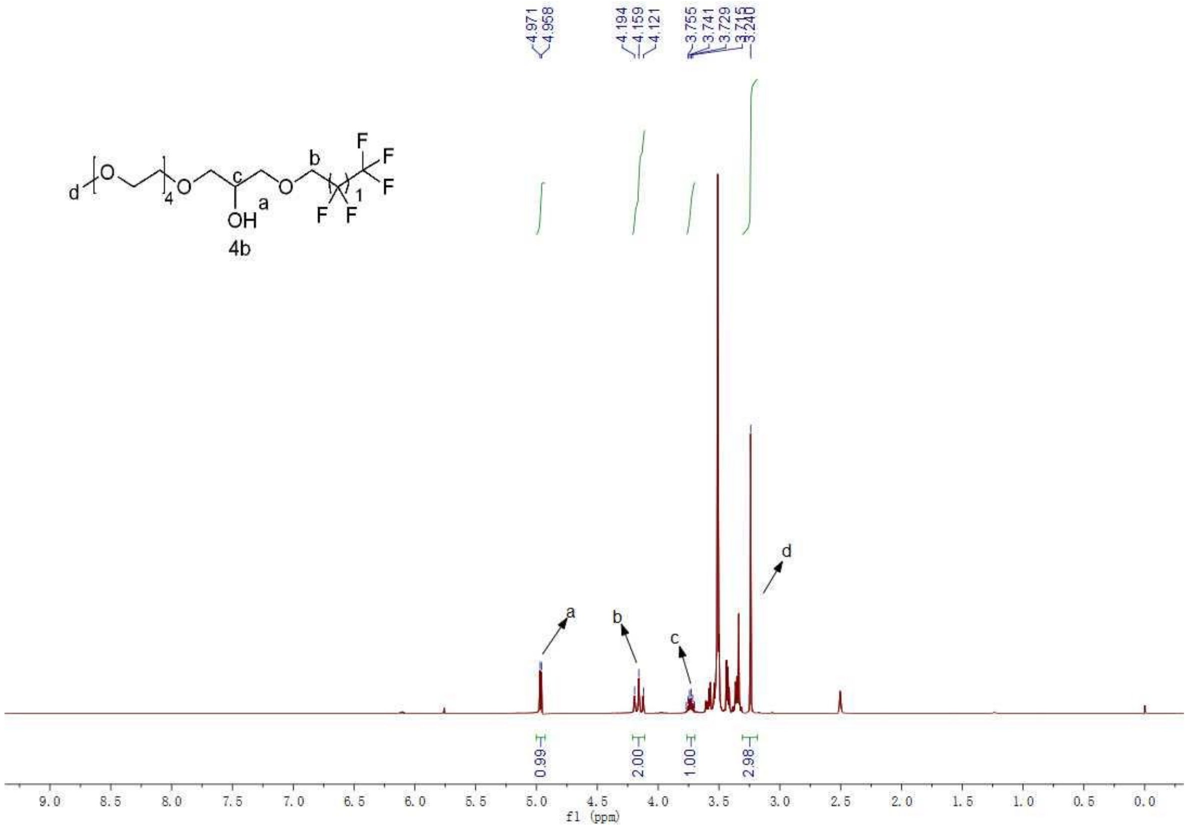

Figure S1. ^{1}H NMR of 4b.

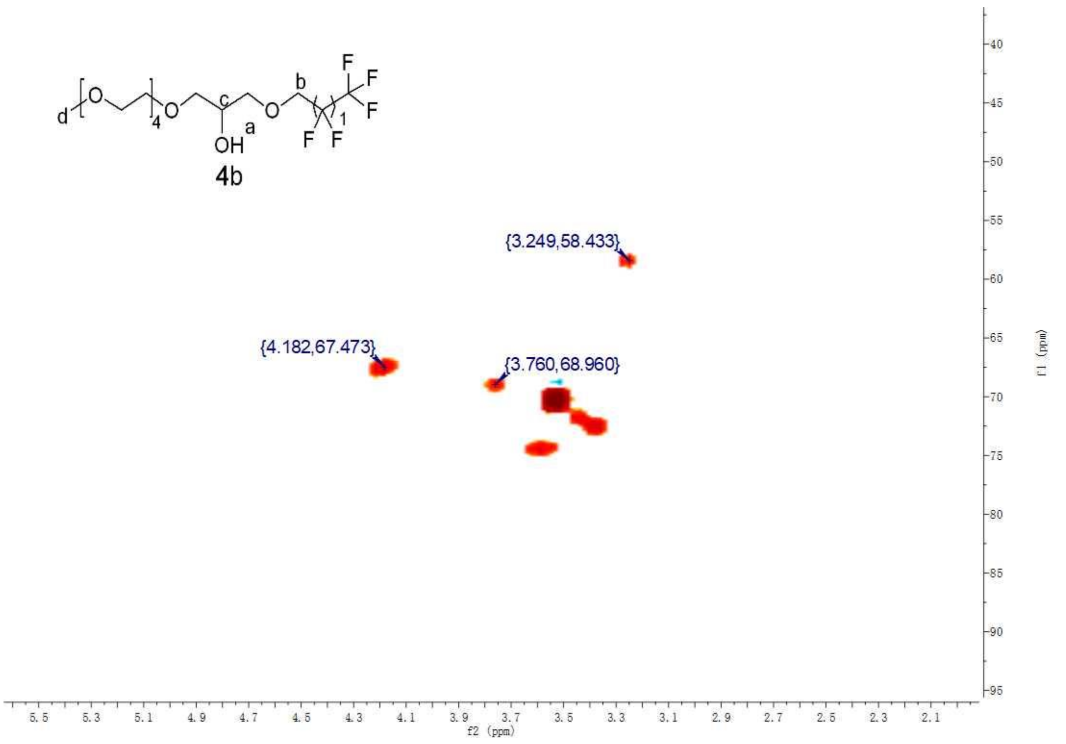

Figure S2. HSQC spectrum of 4b.

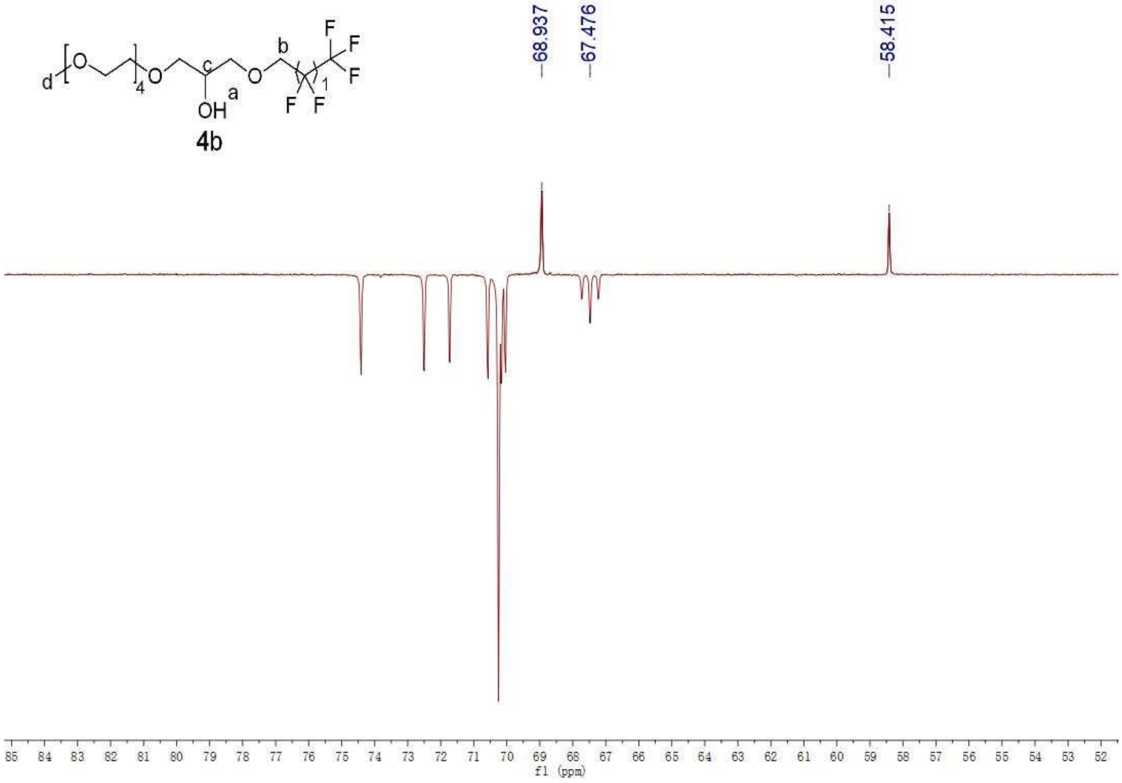

Figure S3. Dept 135 spectrum of 4b.

In the ^{1}H NMR spectrum of **4b** (Figure S1), we confirmed H(d) at δ 3.24. According to the HSQC spectrum (Figure S2), it can be determined that the shift of C connected to H(d) is about 58 ppm. Then based on the Dept 135 spectrum (Figure S3), it can be determined that the chemical shift of C connected to H(c) is 68.937 ppm. Finally, from Figure S2, the chemical shift of H(c) is determined to be 3.76 ppm.

5.1. H NMR and ¹⁹F NMR spectra of fluorinated surfactants (4a, 4b, 6a-6d)

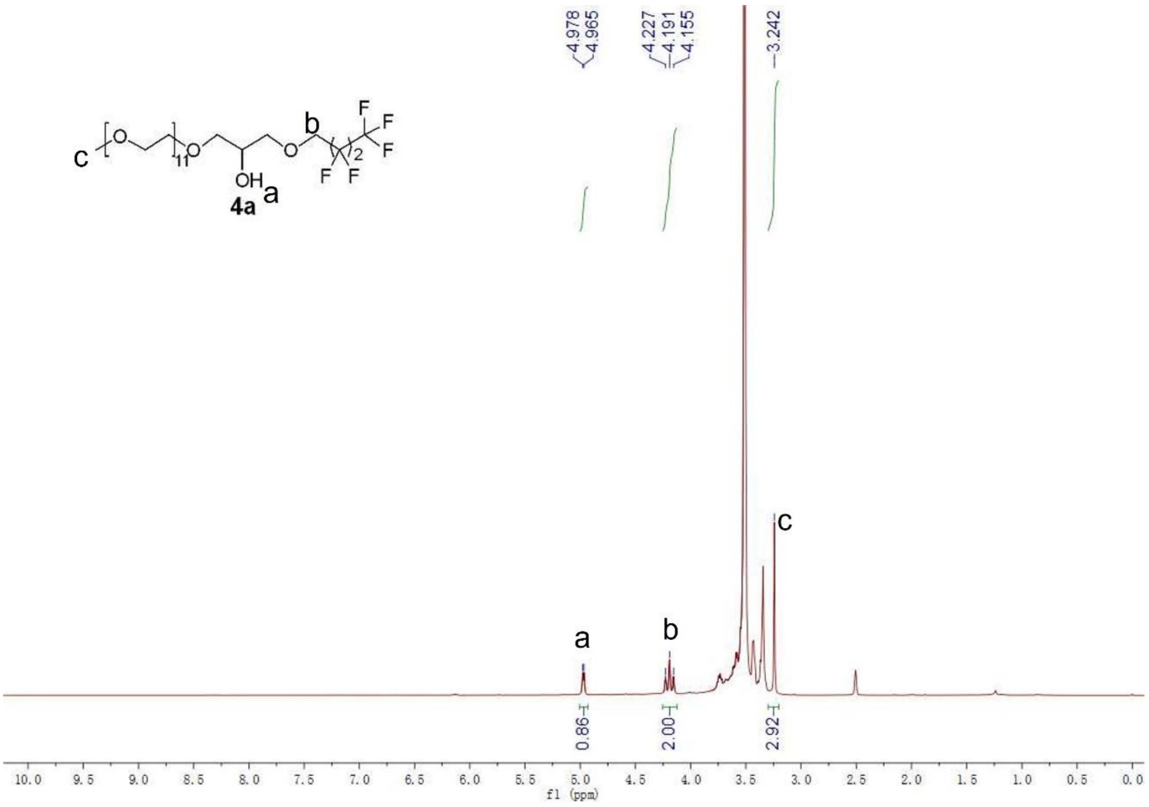

Figure S4. ^{1}H NMR of 4a.

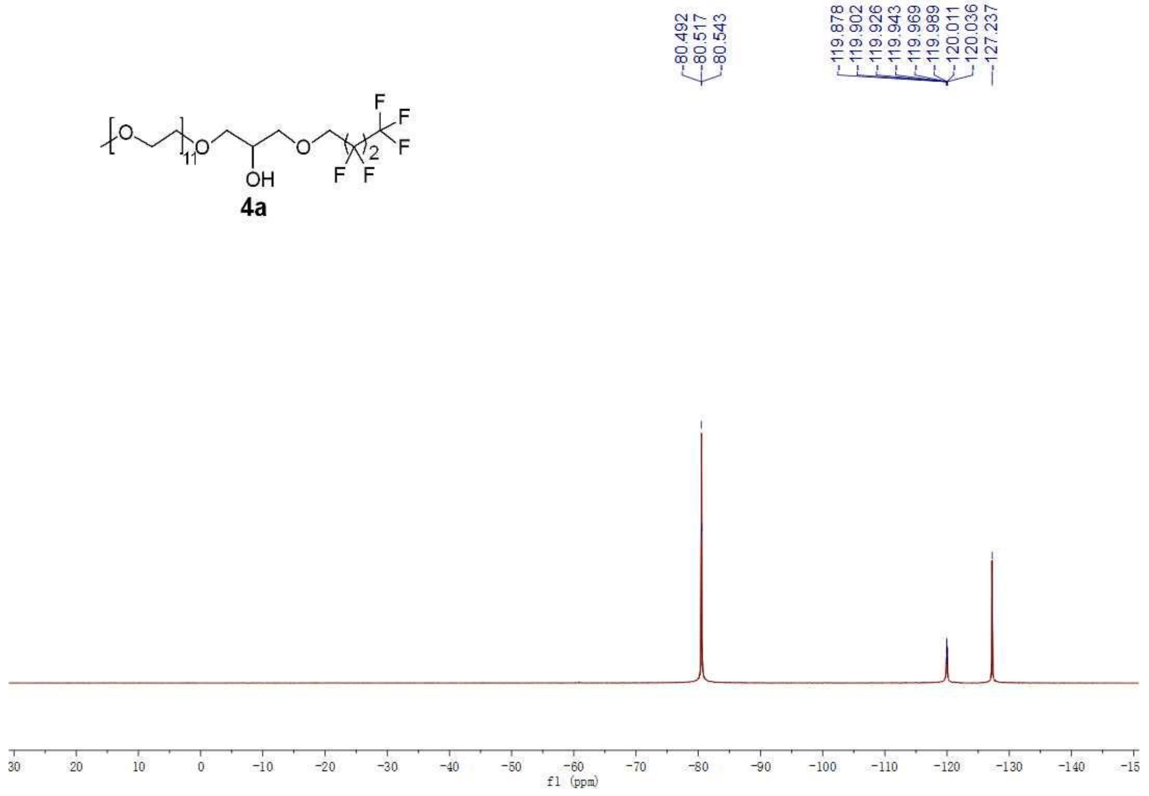

Figure S5. ^{19}F NMR of 4a.

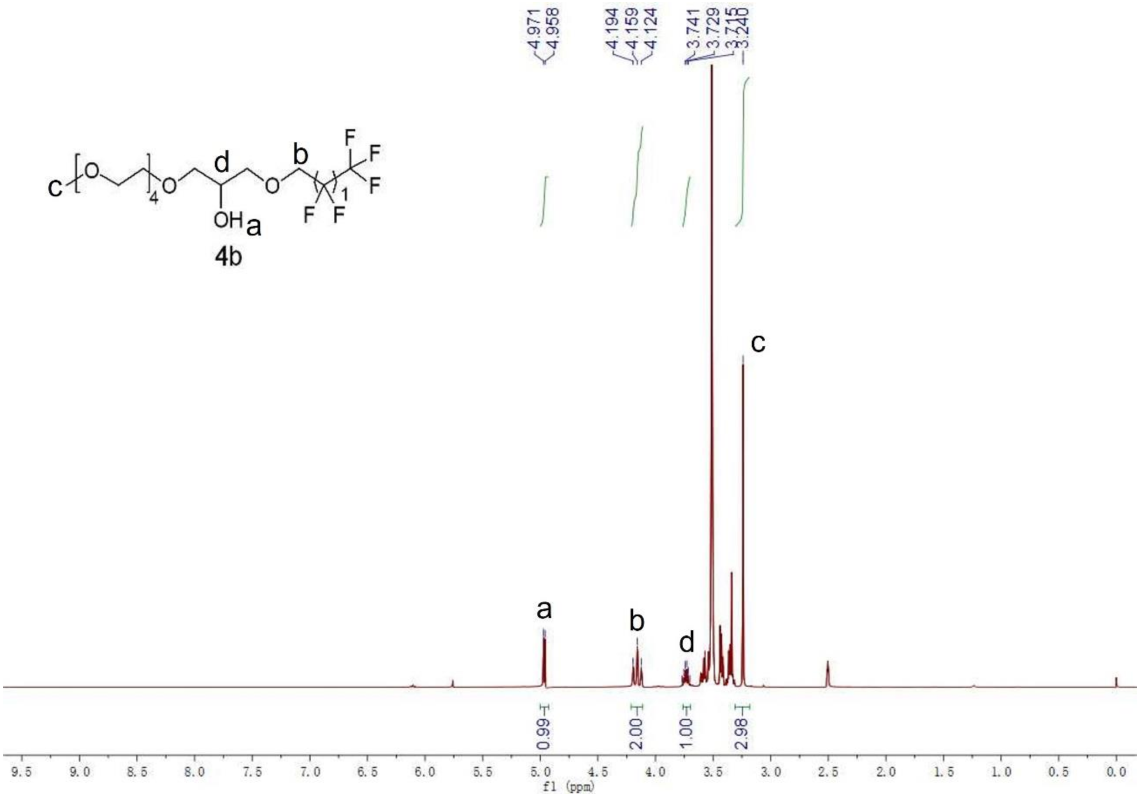

Figure S6. ^{1}H NMR of 4b.

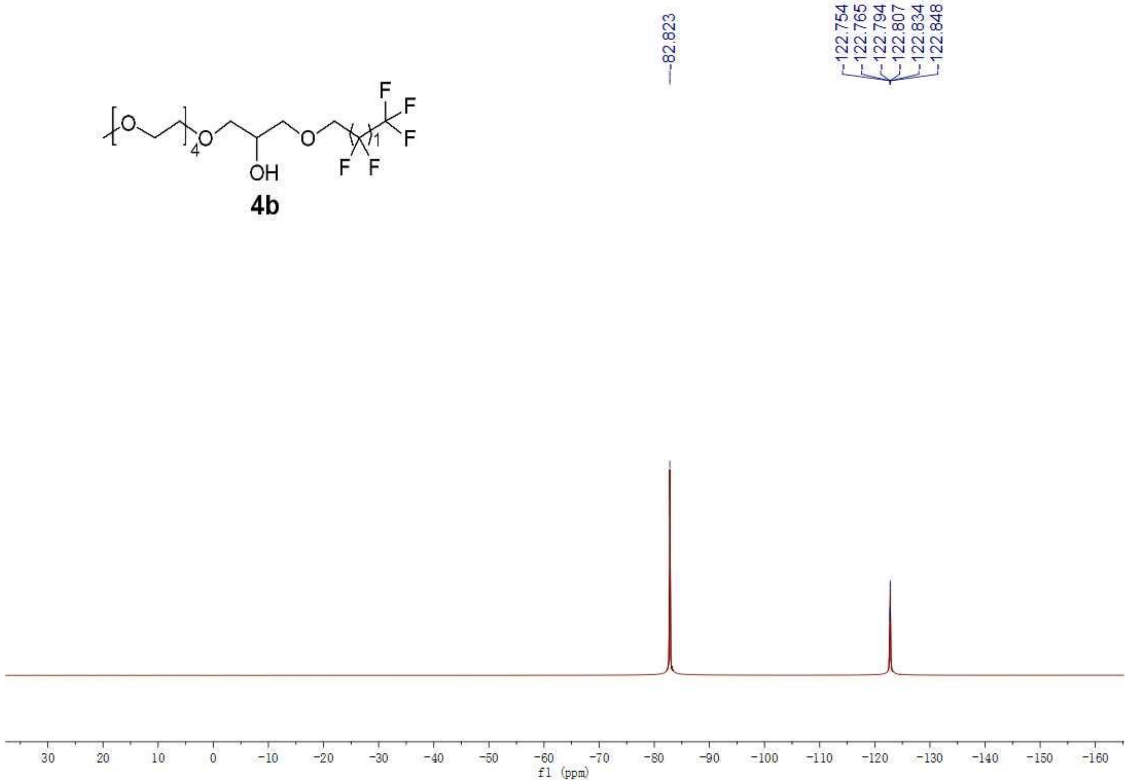

Figure S7. ^{19}F NMR of 4b.

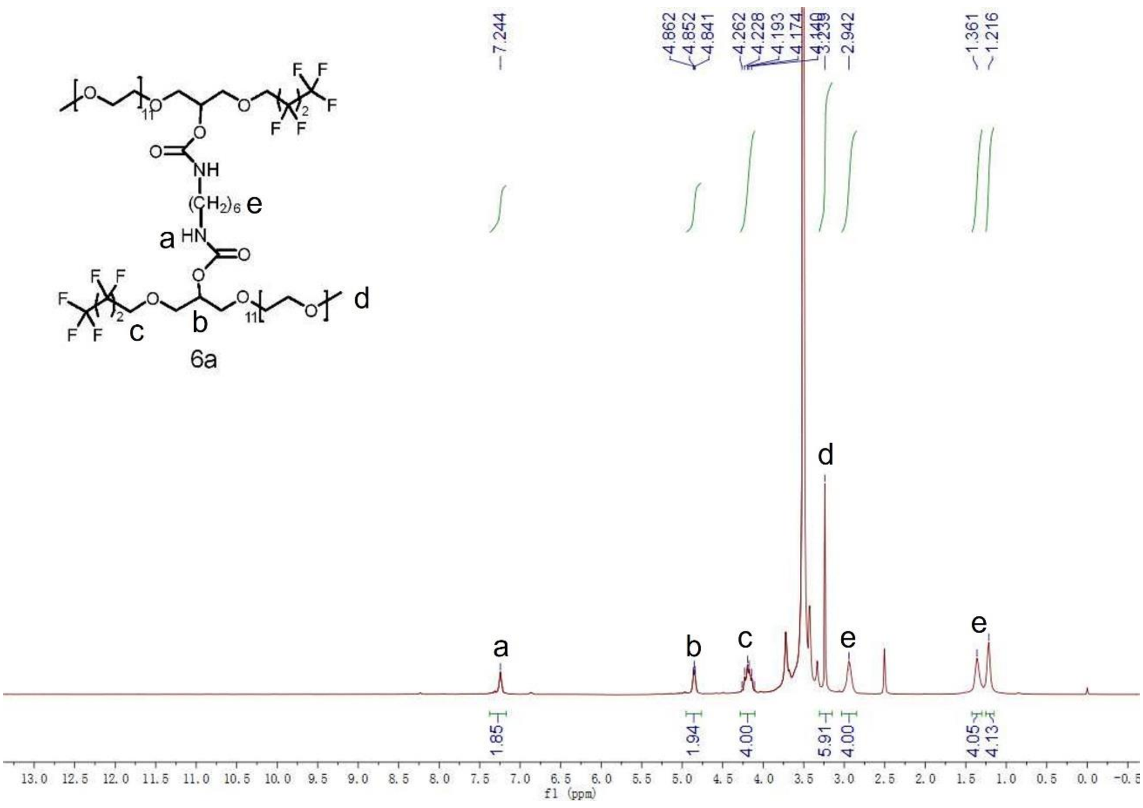

Figure S8. ^{1}H NMR of 6a.

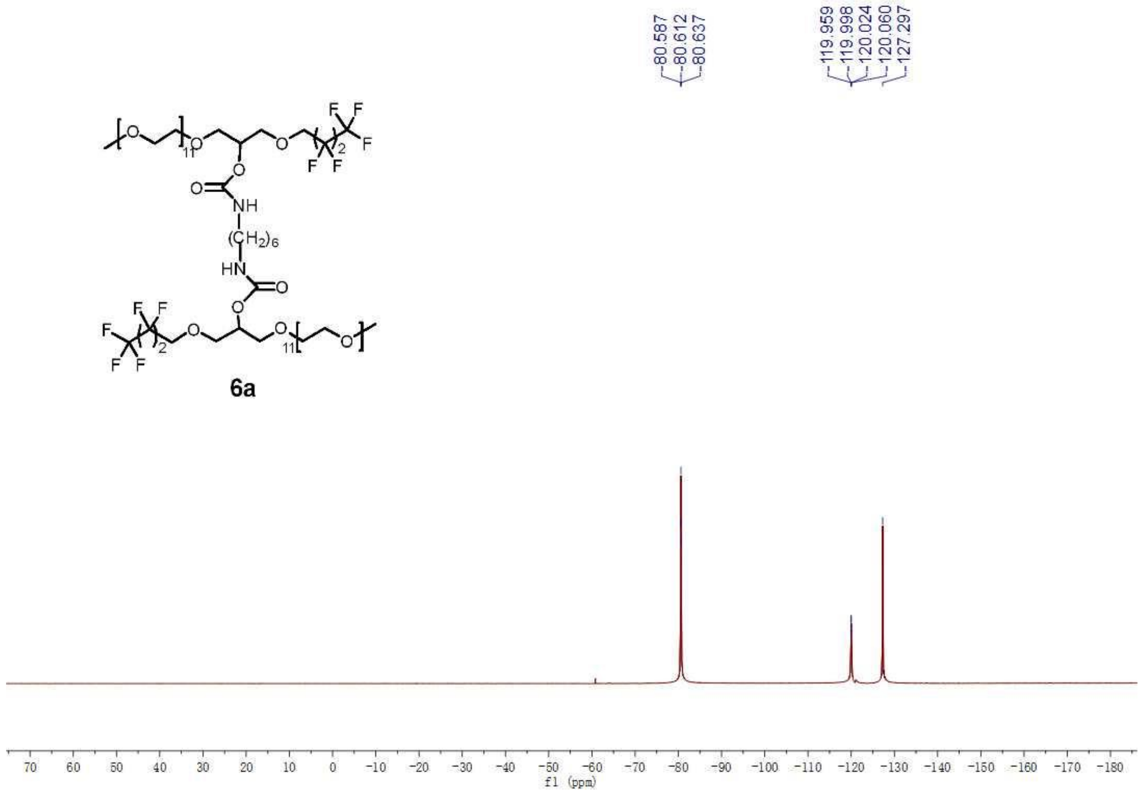

Figure S9. ^{19}F NMR of 6a.

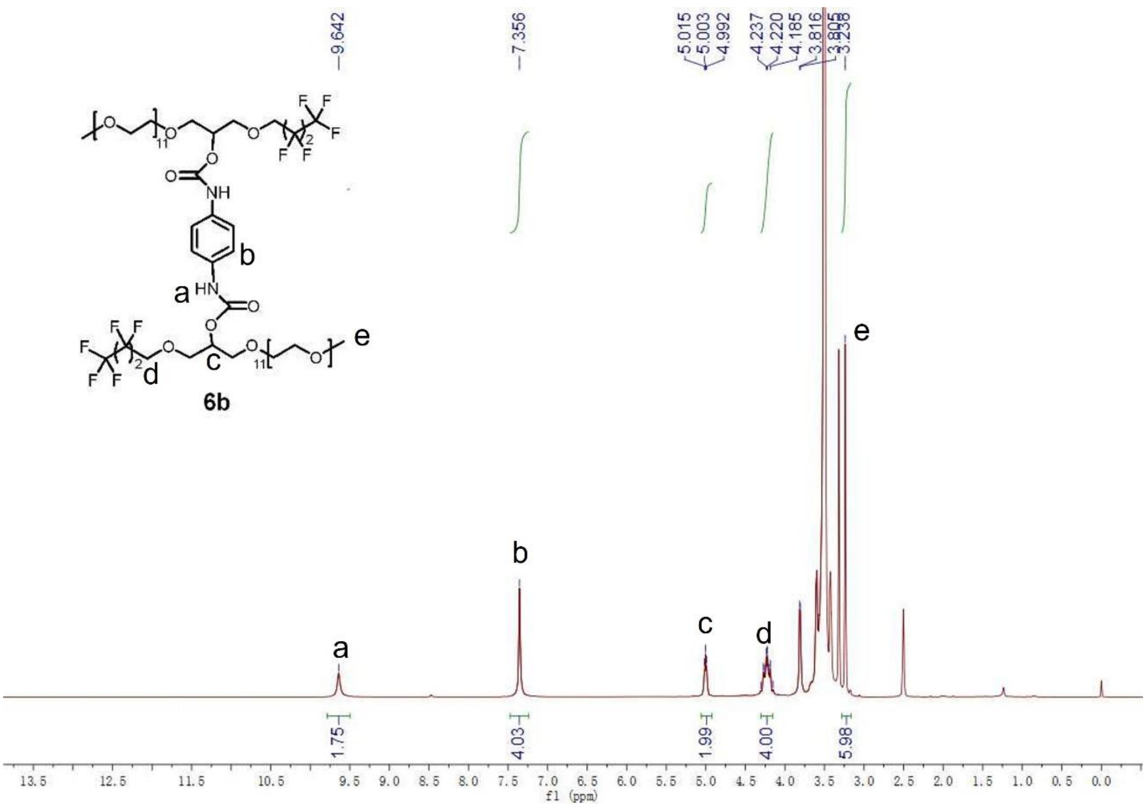

Figure S10. ^{1}H NMR of 6b.

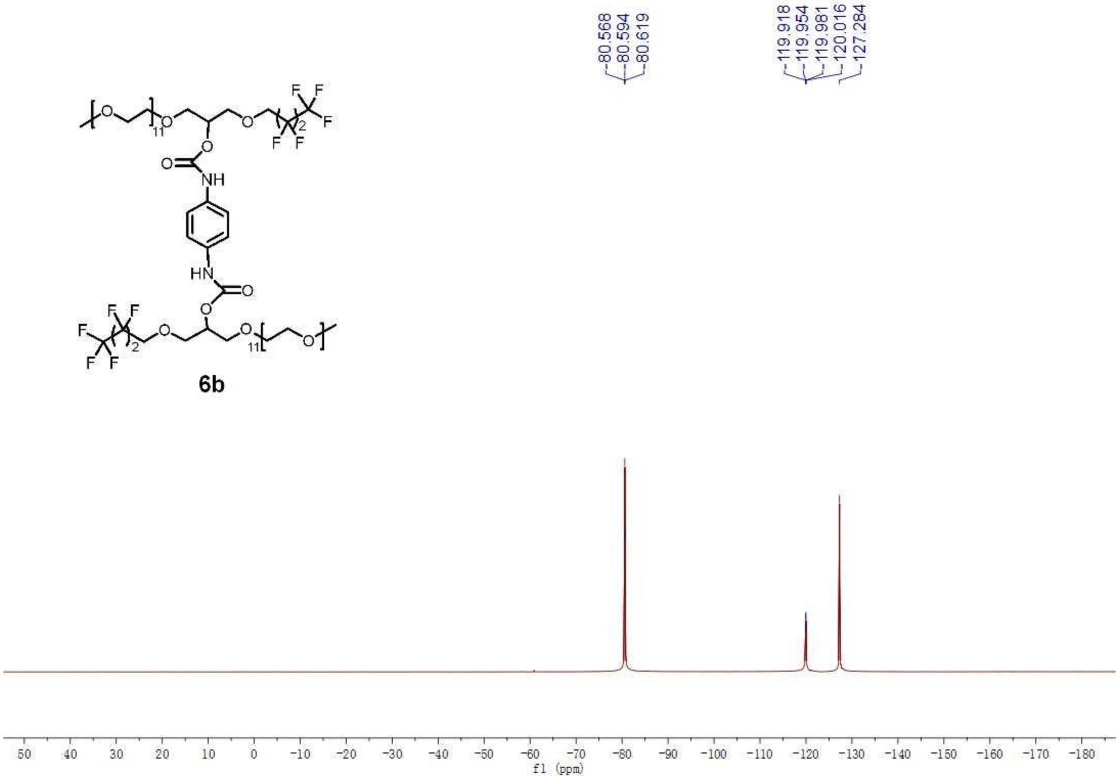

Figure S11. ^{19}F NMR of 6b.

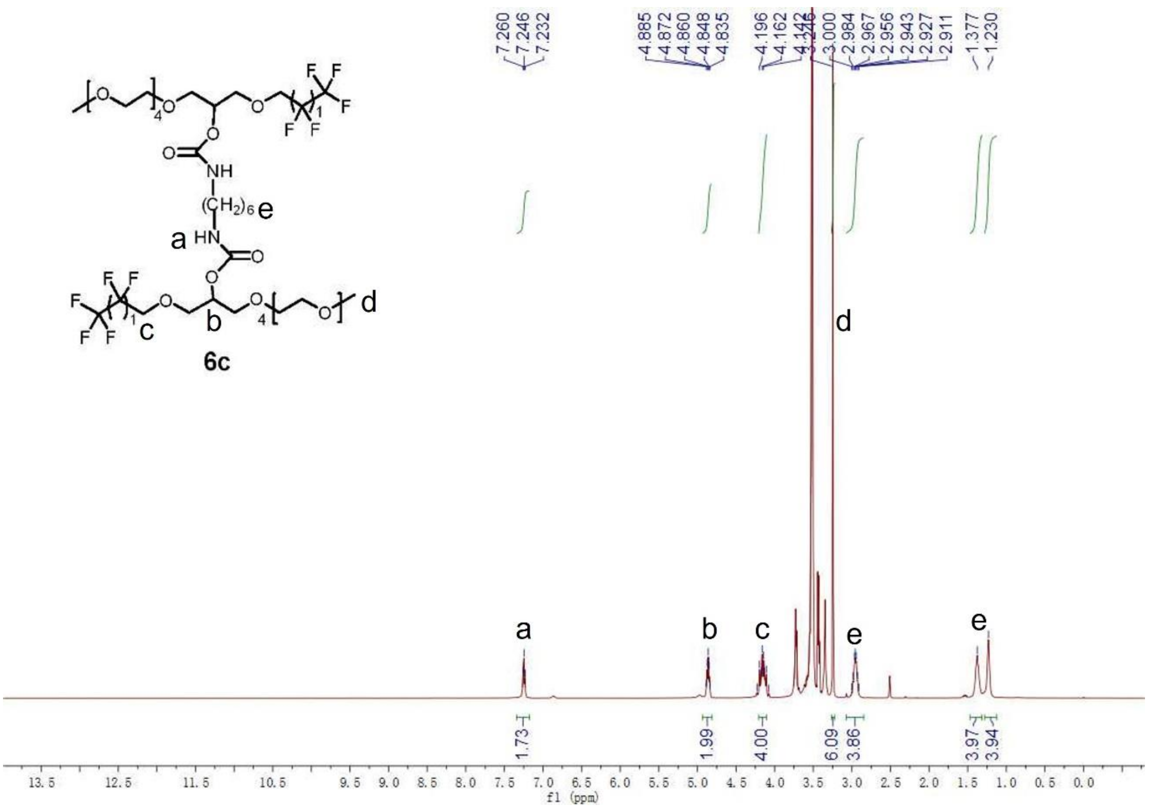

Figure S12. ^{1}H NMR of 6c.

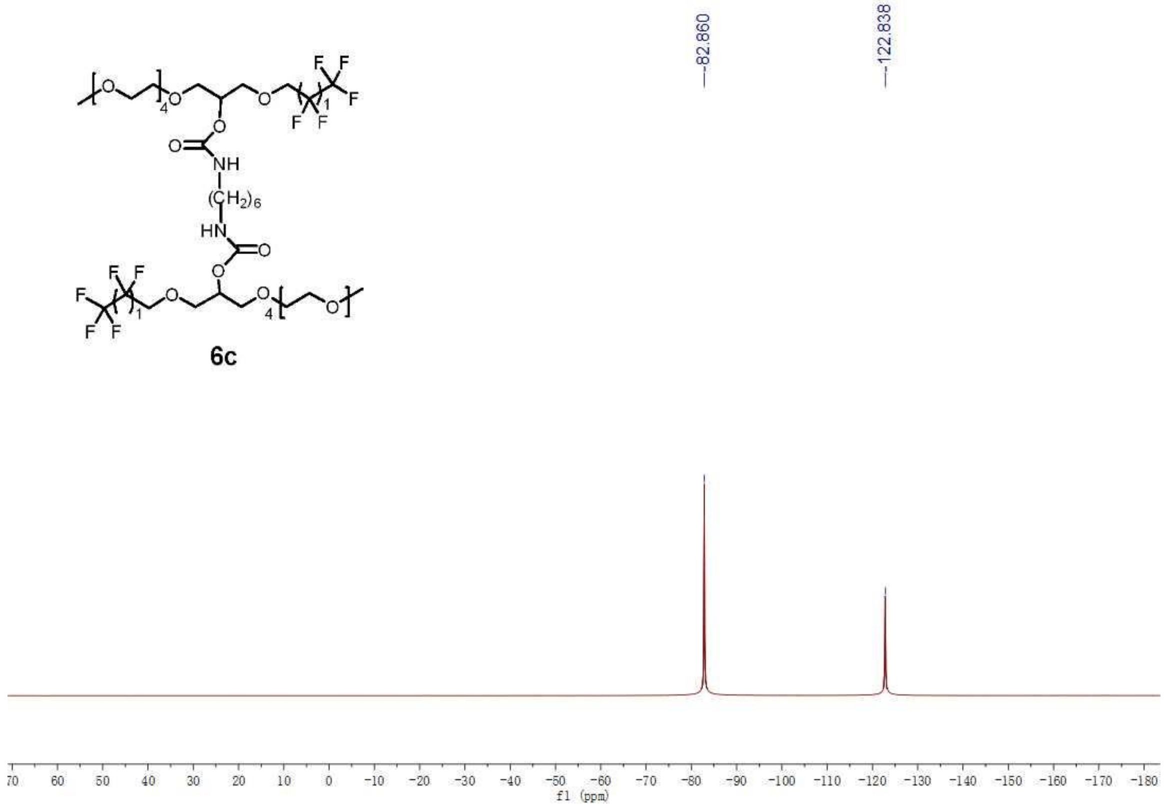

Figure S13. ^{19}F NMR of 6c.

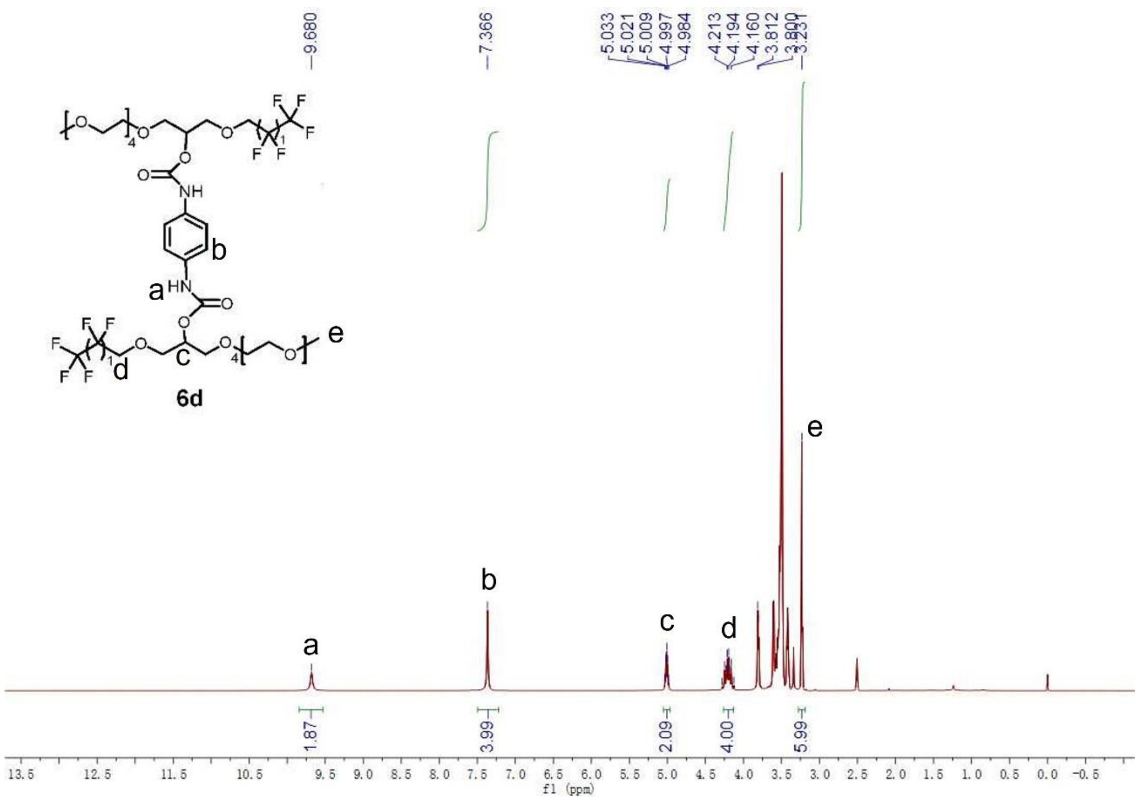

Figure S14. ^{1}H NMR of 6d.

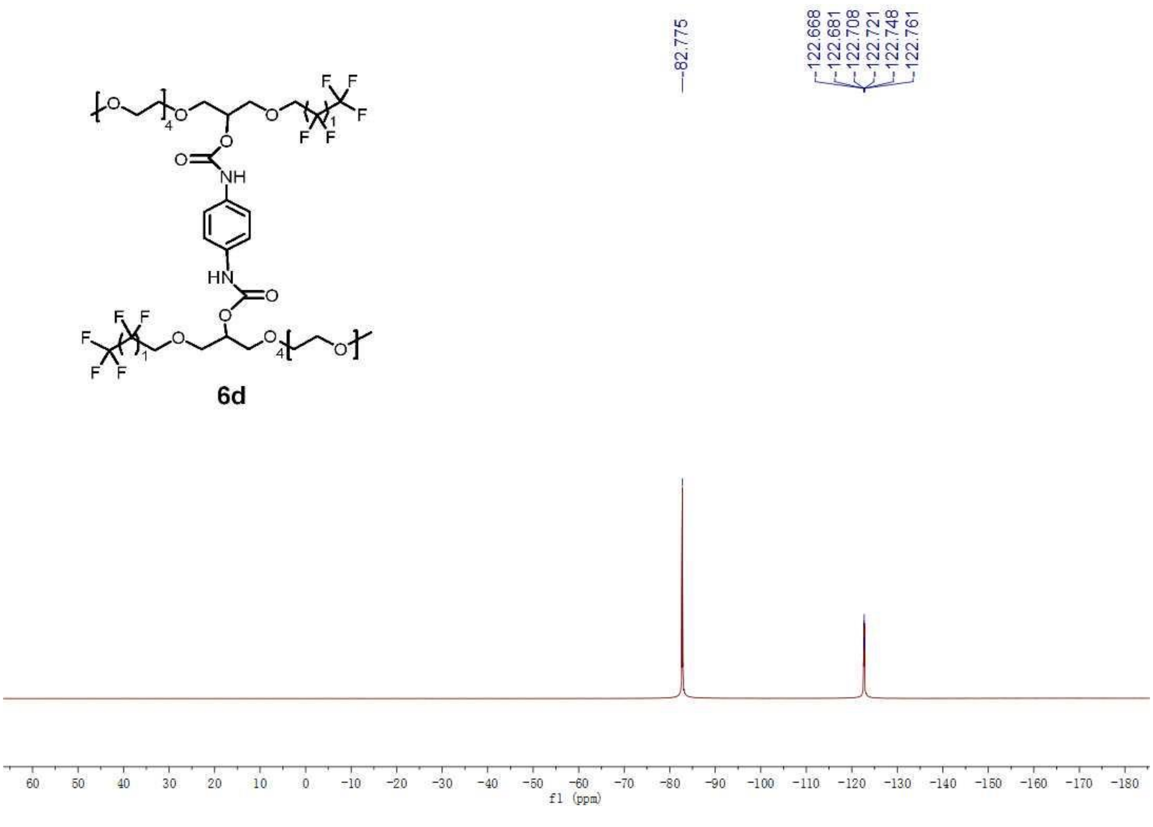

Figure S15. ^{19}F NMR of 6d.

6. References.

1. Wang R. G.; Song L. Y.; Guo Y. Q.; Kou J. J.; Song H. J.; Liu Y. X.; Zhang J. J.; Wang Q. M. Synthesis and structure–activity relationships of nonionic surfactants with short fluorocarbon chains. *J. Mol. Liq.* 2021, 321, 114486.
